# Glomus Jugulare Presenting with Isolated Facial Nerve Palsy

**DOI:** 10.1155/2014/514086

**Published:** 2014-01-02

**Authors:** Angelica A. Nunez, Luis R. Ramos-Duran, Albert C. Cuetter

**Affiliations:** ^1^Department of Neurology, Texas Tech University Health Sciences Center, Paul L. Foster School of Medicine, El Paso, Tx 79905, USA; ^2^Department of Radiology, Texas Tech University Health Sciences Center, Paul L. Foster School of Medicine, El Paso, Tx 79905, USA

## Abstract

Glomus jugulare is a rare slow growing tumor occurring within the jugular foramen that rarely presents with isolated symptoms. Although histologically benign, these tumors are locally destructive because of their proximity to the petrous bone, the lower cranial nerves, and the major vascular structures (Miller et al. (2009) and Silverstone (1973)). We wish to report a glomus jugulare tumor eroding the petrous bone and producing an ipsilateral peripheral facial weakness. The mechanism of this erosion is discussed.

## 1. Case Report

A 73-year-old Caucasian woman, with a history of Parkinson's disease and breast carcinoma, presented with a 2-month history of a rapidly progressive right total facial palsy, hyperacusis, and changes in taste. She had no sensory symptoms.

She had a 7-year history of Parkinson's disease treated with carbidopa 25/100 mg 4 times a day and ropinirole 3 mg tid. She was functional with a Hoehn-Yahr staging of 2, and she was 90% independent. She had a history of breast cancer treated with breast resection and radiation therapy 12 years prior to the presentation. There had been no cancer relapse. She had a brain MRI without gadolinium 3 years prior to the presentation showing mild volume loss of the brain.

On physical examination, she had normal pupils and eye movements. She had weakness of the occipitofrontalis, corrugator supercilii nasalis, risorius, buccinators, zygomaticus major, mentalis, and platysma muscles of the right side of the face. Corneal reflex was decreased on the right. The Weber test lateralized to the left. The Rinne test revealed that air conduction was better than bone conduction bilaterally. There was a low frequency conductive hearing loss of the right ear tested with a 128 HZ tuning fork. Palate and tongue were in the midline. Because of her Parkinsonism, her gait was slow with narrow strides and short steps and the arm swing was reduced. Cerebellar tests were slow but well performed. Muscle stretch reflexes were present and equal. There were no pathologic reflexes.

There was an assumption that she had had a right Bell's palsy and was treated as such. Unenhanced MRI of the brain was reported as normal. As time passed, the facial palsy and hearing deficit did not resolve. There were times where her face was more symmetric than others but her ability to close the right eye had improved.

Three months after presentation, her facial palsy worsened again and she complained of hearing loss in the right ear. Otologic examination showed bulging right tympanic membrane consistent with right otitis media, but examination by the otolaryngologists was negative for middle ear disease. Pure tone audiograms showed a low frequency conductive hearing deficit. A blink reflex was abnormal in the right facial nerve regardless the site stimulated. Direct stimulation of the right facial nerve in front of the ear showed only a slight reduction of amplitude of the compound muscle action potential recorded in facial muscles, compared with the left. This finding with abnormal blink response indicated that the lesion of the facial nerve was in the proximal segment of the nerve.

**Figure 1:**
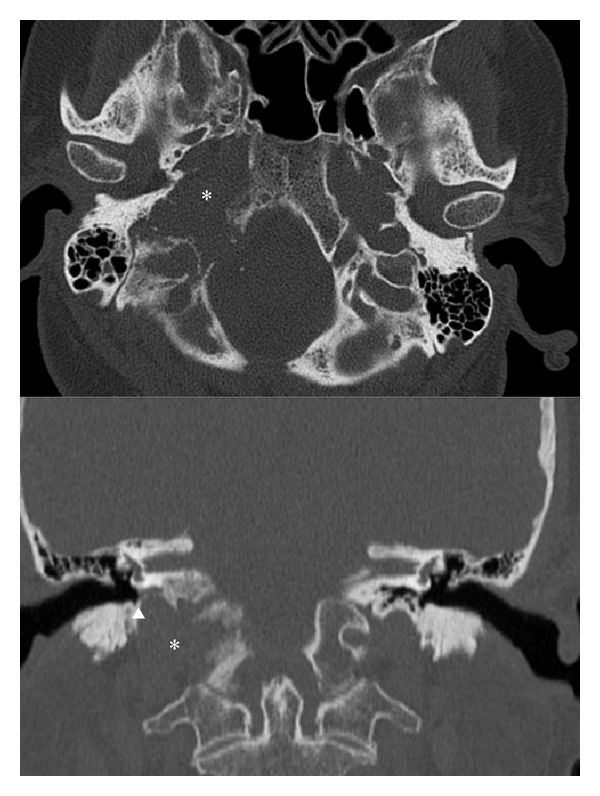
Axial and coronal nonenhanced CT scan reformations using bone reconstruction algorithm demonstrates an osteolytic skull base lesion centered within the right jugular foramen (∗) the lesion exhibits irregular margins with preservation of the ipsilateral hypotympanic sigmoid plate (arrowhead) and without macroscopic extension into the ipsilateral middle ear.

**Figure 2:**
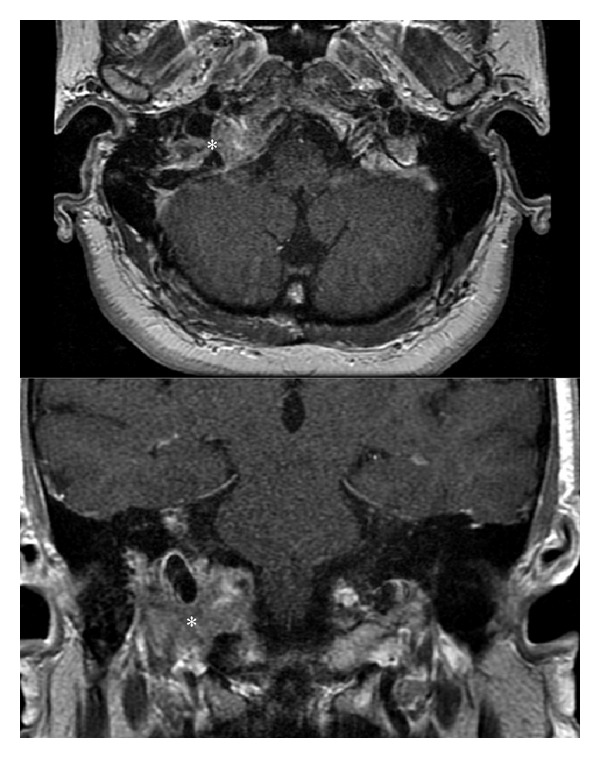
Axial and coronal contrast enhanced T1 MR images demonstrates a solid enhancing tumor at the right jugular foramina (∗) the lesion exhibits heterogeneous internal signal intensity given by the presence of internal flow-voids, resulting in the so-called “salt and pepper” appearance.

A computerized tomogram of petrous bones showed an ill-defined osteolytic process involving the right occipital bone and possible tip of the petrous bone consistent with either metastatic disease or multiple myeloma (Figure 1). There was no evidence of cholesteatoma. A bone scan showed abnormal foci in the right skull base, left calvarium, and mid cervical spine suspicious for malignancy. An MRI with contrast of brain found an enhancing mass dorsal to right jugular vein as it courses through the jugular foramen of the skull base with enhancement characteristics compatible with glomus jugulare (Figure 2). The brain MRA showed only minimal luminal narrowing of right internal carotid. A Doppler ultrasound of the carotid arteries showed no significant stenosis of the right internal carotid artery. An extensive work up for metastatic disease including positron emission tomography was negative. She had a negative workup for multiple myeloma. A CT guided biopsy at level of C5 showed no evidence of malignancy.

The patient then received base of skull radiation therapy, which helped to resolve the facial palsy as well as the hearing loss. She continues to be doing well presently, without further episodes of facial palsy after radiation therapy. The patient declined further treatment of tumor with surgery or radiosurgery. A year after presentation, she has partially recovered the function of the right facial muscles with significant synkinesis of the right orbicularis oculi muscle. A blink reflex showed activity in other right facial muscles denoting reinnervation activity.

## 2. Anatomical Explanation

The jugular foramen is a large aperture in the base of the skull and it is formed anteriorly by the petrous portion of the temporal bone and behind by the occipital bone. It has [1] compartments from front to back that contain the inferior petrous sinus, the cranial nerves IX, X, XI, and the sigmoid sinus (which becomes the internal jugular vein), respectively.

## 3. Discussion

Glomus jugulare is a rare slow growing tumor that occurs within the jugular foramen of the temporal bone. It is a subset of tumors known as paragangliomas, which are tumors that arise from neural crest cells associated with autonomic ganglia. Due to the anatomical location of glomus jugulare within the jugular foramen, growth of this tumor typically involves CN VII, IX, X, XI, which are in the vicinity of the tumor [1–4].

Glomus jugulare usually occurs later in life (60 or 70s) but can appear at any age. Although no known risk factors have been recognized in glomus jugulare tumors, a pathogenic mutation in the gene for the succinate dehydrogenase enzyme has been implicated in the disease. [5] Symptoms typical of the mass effect from the tumor include dysphagia, dizziness, hearing problems or loss, hearing pulsations in ear, hoarseness, pain, and facial palsy.

The tumor is able to compress and affect the facial nerve due to the proximity of the jugular foramen to the path of the nerve [1, 6]. The facial nerve travels into the internal acoustic meatus through the facial canal then out through the stylomastoid foramen. When a tumor in the jugular foramen grows proximally, it affects the vertical/mastoid segment of the facial canal carrying the facial nerve which causes compression and compromises function, as was have seen with our patient. Remarkably, although facial palsy is listed as a presenting symptom of glomus jugulare, most of the literature published describes facial palsy as a side effect of the surgical treatment rather than being the initial symptoms [3, 7]. The few articles that describe facial nerve palsy as being a presenting symptom describes the palsy as being present with a conglomerate of other symptoms due to CN IX, X, XI involvement [1, 3]. Facial palsy due to invasion of the facial canal has rarely been seen as the primary isolated symptom of glomus jugulare [1, 6]. A literature search showed only a few articles mentioning the initial symptoms of these tumors and they are usually described as a combination of pulsatile tinnitus, hearing loss, facial nerve paralysis, vertigo, otalgia, dysphagia, hoarseness, throat sore, episodic hypertension with headaches, and tachycardia [3]. According to one study, facial nerve palsy is only seen in 3% of the cases of glomus jugulare and is rarely the sole symptom. Another article also describes the usual presenting symptoms as conductive hearing loss and pulsatile tinnitus and attributes growth of the tumor to later causing the remaining constellation of symptoms (facial nerve paralysis, vertigo, hoarseness, and paralysis of lower cranial nerves) [3]; unlike the patient presented here where hearing loss occurred months after the onset of facial paralysis. Interestingly a common physical finding in glomus jugulare tumors is a retrotympanic mass often accompanied with bulging tympanic membrane [3], as was seen in our patient; however, this finding did not appear until after her facial palsy indicating that the mass affected her facial nerve primarily and then affected her middle ear function subsequently. In summary, this is a unique presentation of glomus jugulare in which isolated facial nerve palsy was the primary clinical presentation and with a delayed presentation of hearing difficulties. Although these tumors are rare causes of facial nerve palsy, they should be included in the differential diagnosis of total facial nerve palsy [6, 8].
